# Structured Virtual Reality Exergame Intervention in Adolescents With Mild Intellectual Disabilities: Pre-Post Pilot Study on Motor Proficiency and Reaction Time

**DOI:** 10.2196/95572

**Published:** 2026-08-14

**Authors:** Julia Ciążyńska, Agnieszka Kazimiera Rudowicz, Aneta Worska, Janusz Maciaszek

**Affiliations:** 1 Department of Physical Activity and Health Promotion Science, Poznań University of Physical Education, Królowej Jadwigi 27/39, Poznan, Poland, 48 (61) 835 50 07; 2 Special School Complex No. 102, Poznan, Poland

**Keywords:** active video game, bilateral coordination, exergame, gross motor movements, health promotion, intellectual disabilities, physical activity, physical education, postural stability, virtual reality

## Abstract

**Background:**

Adolescents with mild intellectual disabilities often exhibit deficits in motor proficiency and neurocognitive processing, which can limit their functional independence. Although virtual reality (VR) exergames provide a motivating platform for physical activity, objective data quantifying their impact on standardized motor scales remain scarce, particularly when implemented through structured pedagogical frameworks. Earlier research established the WISH (warm-up, imitation, settings, half-hour) and WON (warm-up, objective evaluation, no problem!) protocols to facilitate gameplay independence in this population. However, the extent to which this systematic approach translates into measurable clinical motor and reaction time gains requires further investigation.

**Objective:**

This study evaluated the effectiveness of a 16-session structured VR exergame intervention, using the WISH and WON protocols, in enhancing motor proficiency and shortening simple reaction time among adolescents with mild intellectual disability.

**Methods:**

A multisession, single-group pretest and posttest pilot design was used. A convenience sample of 8 adolescents (3 males, 5 females; mean age 17.63, SD 1.77, range 15‐20 years) was recruited from a special school complex in Poland. The intervention was conducted over an 8-month period (October 2023 to May 2024), consisting of 16 sessions conducted once every 2 weeks. The rhythmic fitness game “OhShape!” (Odders Lab) was played on the Meta Quest 2 system. Motor proficiency was assessed with the Bruininks-Oseretsky Test of Motor Proficiency short form, and simple reaction time to visual and auditory stimuli was measured using a standardized Alfa-Electronics meter. Statistical analysis was performed using the Wilcoxon signed-rank test (α=.05).

**Results:**

Total Bruininks-Oseretsky Test of Motor Proficiency scores significantly improved from a baseline mean of 69.25 (SD 6.80; 95% CI 63.56‐74.94) to 80.50 (SD 5.50; 95% CI 75.90‐85.10) at the posttest (*P*=.01; *r*=0.74). Based on the standardized 5-point proficiency scale, the group mean rose from 1.88 (SD .64; 95% CI 1.34‐2.42) at baseline to 2.88 (SD .35; 95% CI 2.59‐3.17) postintervention (*P*=.008; *r*=0.89). Furthermore, the mean simple reaction time decreased from 426.50 (SD 52.3; 95% CI 382.78‐470.22) ms to 392.10 (SD 38.4; 95% CI 359.98‐424.22) ms at the final assessment (*P*=.02; *r*=0.72).

**Conclusions:**

This pilot study suggests structured VR exergaming may address motor deficits in adolescents with mild intellectual disability. Promoting autonomy via protocols was associated with measurable motor gains within this cohort. Although the absence of a control group limits causal claims, immersive VR may offer a supportive tool for neuro-motor adaptations in special education, warranting validation in larger controlled trials.

## Introduction

### Background and Rationale

Intellectual disability (ID) is characterized by significant limitations in intellectual functioning and adaptive behavior [1]. Children and adolescents with ID are more vulnerable to obesity and secondary metabolic health issues than typically developing children [2,3]. Beyond cardiovascular and metabolic concerns, individuals with mild ID frequently exhibit impaired motor proficiency, including delays in gross motor movements, bilateral coordination, and postural stability [4-6]. Consequently, this population often presents with strikingly low health-related physical fitness levels [7], and their physical inactivity is a major contributor to poor health and reduced quality of life [8]. While traditional sports games and structured athletic programs have been shown to effectively improve motor skills, coordination, and overall physical fitness in youth with ID [9], immersive virtual reality (VR) exergames have emerged as a transformative, complementary solution. These digital platforms offer distraction-free environments that foster higher exercise intensities and greater persistence [10,11]. However, a significant problem persists: despite the potential of VR, many interventions focus primarily on participation metrics rather than objective clinical outcomes [12,13]. Relying solely on subjective engagement metrics or in-game scores is insufficient to confirm real-world functional transfer. Therefore, using rigorous, standardized clinical assessments is critically needed to scientifically validate whether digital gameplay translates into genuine neuro-motor adaptations and tangible developmental progress for the ID population.

### Review of Relevant Scholarship

Recent advancements suggest that immersive serious games act as holistic educational environments, facilitating the training of meta-skills, cognitive, emotional, and behavioral, which are crucial for functional adaptation [14]. Specifically, gamified interventions have demonstrated a significant positive impact on problem-solving abilities and concept development in students with ID, bridging the gap between digital play and cognitive growth [15]. Educational and inclusive environments have also been shown to further support these developments, positively influencing fine motor skills and attention levels [16]. Furthermore, recent meta-analyses indicate that the benefits of digital technology-based interventions for neuro-cognitive efficiency are not universally consistent, often depending on the specific cognitive domains targeted, the clinical characteristics of the population [17-19], as well as the recruitment settings and delivery contexts used [20].

However, individuals with ID demonstrate significant learning curves and adaptive gains in digital environments, suggesting that immersive VR can facilitate real-world skill transfer if properly structured [21,22]. This is supported by findings that while VR exergaming effectively improves locomotor skills in children with developmental disabilities, it is insufficient on its own to significantly enhance object control skills or overall physical activity levels, indicating that VR must be mixed with reality-based programs [23]. Crucially, the latest longitudinal evidence strongly confirms that while fully immersive VR enhances cognitive-motor skills during gameplay, their transfer to specific real-world tasks and generalized response times remains critically limited without integration into practical, structured training frameworks [24]. To maintain long-term adherence and intrinsic motivation, serious games must incorporate optimal challenges and be matched to the player’s behavior [25]. Furthermore, recent qualitative research highlights that carefully calibrated immersive VR environments, when combined with facilitative, nondirective professional support, serve as psychologically safe platforms that can strengthen self-determination, autonomy, and action-control beliefs in adults with ID [26]. The lack of structured instructional protocols has been a significant barrier to the effective application of VR in this population. Evidence from various motor skill interventions [27], including object control skills training [28], highlights the importance of systematically designed, error-reduced approaches. Previous work addressed this by establishing the WISH (warm-up, imitation, settings, half-hour) and WON (warm-up, objective evaluation, no problem!) training protocols, which facilitate gameplay independence [29]. Recent innovations in telehealth and mHealth support systems further demonstrate that remote, technology-aided interventions can effectively stimulate physical activity, leisure engagement, and mobility for individuals with ID and related mobility disabilities [30-32]. While earlier reports suggest these protocols are effective for fostering autonomy [29], the current report differs by shifting the focus from subjective independence metrics to the evaluation of objective clinical outcomes. Evaluating the efficacy of immersive therapeutic interventions [33] requires accurately measuring functional gains. Therefore, this study relies on standardized clinical tools, specifically the Bruininks-Oseretsky Test of Motor Proficiency (BOT-2), whose validity and reliability for detecting genuine neuro-motor adaptations in youth with IDs are strongly supported by recent methodological literature [34,35].

### Hypothesis, Aims, and Objectives

The primary aim of this pilot study was to evaluate the feasibility and preliminary effectiveness of a 16-session VR exergame intervention, governed by the WISH and WON protocols, in improving objective motor proficiency and shortening simple reaction time (SRT) in adolescents with mild ID. To foster intrinsic motivation and ensure sustained engagement, the core expectations and instructional frameworks were derived from Self-Determination Theory. This theory emphasizes that teachers’ motivational behaviors, specifically providing autonomy support and competence-building feedback, are vital for pupils’ persistence and internal satisfaction during technology-mediated learning [36].

Given the pilot nature of this study, the hypotheses were formulated to explore preliminary trends and directional changes rather than definitive clinical efficacy. Specifically, this study evaluated the following hypotheses:

- Primary hypothesis: the structured VR intervention was expected to elicit positive shifts in global motor proficiency, as measured by the BOT-2 short form (SF) total point score.
- Secondary hypothesis: the intervention was hypothesized to enhance central cognitive efficiency, which would be reflected in a trend toward shortened SRT to visual and auditory stimuli.

Additionally, secondary exploratory analyses were preplanned to evaluate whether raw numerical point gains on the BOT-2 translated into meaningful qualitative shifts across clinical descriptive categories (eg, moving from “Well Below Average” to “Below Average” or “Average”), and whether the intervention improved intra-individual neuro-cognitive response stability over time.

To appropriately address these objectives, a single-group pretest and posttest pilot design was selected. This specific design relates directly to the exploratory nature of the study by using a within-subject comparison to minimize the confounding impact of high interindividual variability in motor and cognitive baselines, a common characteristic of the ID population. This approach allows for the safe and targeted exploration of potential developmental trends under identical environmental conditions before moving toward larger controlled trials.

## Methods

### Inclusion and Exclusion

Eligibility was determined based on a comprehensive set of criteria to ensure safety and data reliability. To be included, individuals were required to have a formal diagnosis of mild ID and no prior experience with immersive VR technology. Participants had to be current pupils of a special education school, within the age range of 8 to 25 years. Methodological requirements specified that candidates must be efficiently handed (both right and left), not be taking psychotropic medications, and be free from chronic or acute physical injuries. A documented statement from the school psychologist and physical education (PE) instructor was mandatory to confirm that each student possessed sufficient physical and mental health for participation. Students requiring vision correction were eligible provided they used specialized overlays for glasses within the VR headset.

Conversely, individuals were excluded if they had previous exposure to VR environments or did not meet the diagnostic criteria for mild ID. Other exclusion factors included being over 25 years of age, a history of ophthalmic surgery, or current use of psychotropic drugs. History of fresh or long-term injuries also led to disqualification. Finally, candidates were excluded if school staff determined their health status was below the threshold necessary for standard participation in PE lessons.

### Participant Characteristics

A total of 10 eligible students were identified and approached for participation. Of those approached, 100% (8/8) of the individuals and their legal guardians provided informed consent to enroll in the study. Consequently, 10 participants commenced the intervention. However, two participants dropped out during the course of the study due to personal reasons and withdrawal from the school. In accordance with established reporting standards, recruitment was officially closed upon exhausting the available pool of eligible students within the facility for the current academic year, which served as the predefined stopping rule establishing the final cohort size. Thus, the final analytical sample consisted of 8 adolescents (3 males and 5 females). Participant age ranged from 15 to 20 years, with a mean age of 17.63 (SD 1.77) years. The group was considered relatively homogeneous, sharing a formal diagnosis of mild ID. At the developmental stage of late adolescence, participants demonstrated similar baseline motor proficiency levels, as evidenced by their pretest BOT-2 scores.

### Ethical Considerations

The study was approved by the Institutional Review Board at the Karol Marcinkowski Medical University in Poznań (No. 684/23). All procedures adhered to ethical standards, ensuring voluntary participation. No payments or monetary incentives were made to participants. Informed consent was obtained from parents or legal guardians, and verbal assent was secured from the students. All collected data were strictly anonymized, and confidentiality measures were implemented to protect participants’ privacy throughout the research process. To ensure complete nonidentifiability in the visual materials, facial features of the participants were obscured using the PicDefacer image anonymization tool (Barakin Digital). Furthermore, explicit written informed consent was obtained from the parents or legal guardians specifically granting permission for the publication of these anonymized photographs.

### Sample Size, Power, and Precision

As this was a pilot study focusing on the initial efficacy of a structured pedagogical framework in a specialized setting, a formal a priori power analysis was not conducted. The sample size (n=8) was determined by the total available cohort of students at the participating facility who met all eligibility criteria during the study period. Precision of parameter estimates was evaluated through the reporting of 95% CI for all primary motor and cognitive outcomes.

### Measures and Covariates

The primary measures defined and evaluated in this study included global motor proficiency and SRT to visual and auditory stimuli (pooled average across both modalities).

### Data Collection

The intervention used the Meta Quest 2 standalone VR system featuring a wireless head-mounted display (1832 x 1920 pixels per eye; 60‐90 Hz refresh rate) and two 6 degrees of freedom hand controllers. Participants played “OhShape!” (Meta Quest 2; Odders Lab), a rhythmic VR fitness game requiring full-body synchronization with an incoming musical stream within a 2.0 ×2.0 meter safe zone. Core mechanics involved matching precision silhouettes for bilateral coordination, smashing red obstacles with boxing-style punches for upper-limb reaction speed, and performing squats or lateral “step-and-reach” shifts to avoid barriers, thereby training dynamic stability and core strength. To ensure an optimal challenge tailored to the unique learning curves of individuals with ID, difficulty levels were dynamically adjusted based on a 5-star performance system, while treatment fidelity was maintained by monitoring the participant’s real-time perspective via a linked smartphone application.

The sessions were integrated into regular PE classes and supervised by a trained instructor. The 16 sessions were divided into 2 phases based on previously established protocols:

- WISH phase (sessions 1‐4): focused on movement technique, technology familiarization, and reducing anxiety.
- WON phase (sessions 5‐16): focused on individual progress, increasing difficulty levels, and fostering gameplay autonomy.

To ensure sustained engagement, instructor behaviors were operationalized based on the SDT-based classification [26,36]. This involved providing autonomy support by gradually shifting gameplay control to the participant during the WON phase, supporting competence through optimal challenges and structured feedback, and fostering relatedness through supportive instructor involvement and emotional encouragement throughout all sessions.

### Quality of Measurements

To enhance the quality of measurements and ensure methodological rigor, all pretest and posttest assessments were strictly standardized, occurring under identical environmental conditions (same room, time of day, and examiners) using validated instruments. Specifically, the SRT test was performed three times for each participant during each assessment series to enhance measurement reliability, maximize data stability, and minimize intraindividual performance fluctuations.

### Instrumentation

#### Motor Proficiency (BOT-2 SF)

Motor proficiency was assessed using the BOT-2 SF [37]. This standardized instrument is highly effective for populations with developmental challenges, demonstrating excellent internal consistency and superior test-retest reliability specifically in individuals with IDs [38]. The SF version was specifically selected for this study due to its high psychometric efficiency and its strong correlation with the complete form (*r*=0.80-0.87) [39,40].

**Figure 1.**
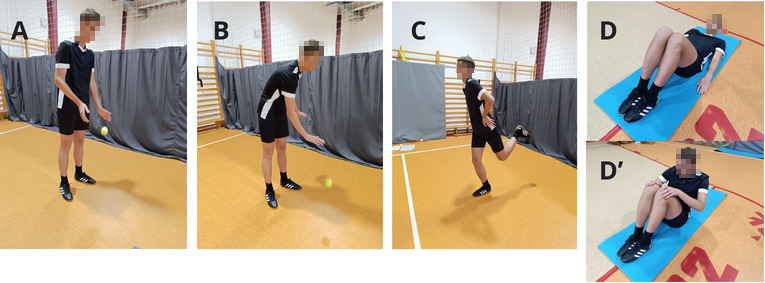
Evaluation of gross motor skills: selected coordination and strength tasks from the Bruininks-Oseretsky Test of Motor Proficiency (BOT-2) Short Form in adolescents with mild intellectual disabilities (n=8) at a special school in Poland (October 2023 to May 2024). Subitems illustrated include: (A) upper-limb coordination (catching a tossed ball); (B) upper-limb coordination (dribbling a ball with an open palm); (C) bilateral coordination (one-leg stationary hop); (D) strength (sit-up initiation phase); and (D’) strength (sit-up completion phase).

**Figure 2.**
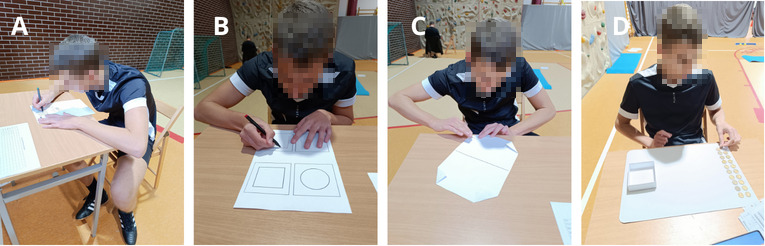
Evaluation of fine motor skills: selected precision, integration, and manual dexterity tasks from the Bruininks-Oseretsky Test of Motor Proficiency (BOT-2) Short Form in adolescents with mild intellectual disabilities (n=8) at a special school in Poland (October 2023 to May 2024). Subitems illustrated include: (A) fine motor precision (drawing lines); (B) fine motor integration (copying shapes); (C) manual dexterity (paper folding); and (D) manual dexterity (transferring pennies).

Crucially, the abbreviated administration time of 15‐20 minutes mitigates the risk of cognitive fatigue and limited attention spans common in youth with mild ID [39,40]. Furthermore, the inclusion of visual supports and photographs in the administration easel minimizes language demands, making the assessment more accessible for participants with receptive language deficits [37,41]. Recent evidence confirms that the BOT-2 is a responsive tool for monitoring psychomotor progress following technological and game-based interventions [41,42]. By using benchmarks such as the minimal detectable change, the BOT-2 serves as an essential resource for monitoring the effectiveness of clinical rehabilitation in children with disabilities [38,42]. The standardized administration of the subtests, conducted in a controlled school environment to ensure ecological validity, is illustrated in Figures 1 and 2.

#### Simple Reaction Time (SRT)

**Figure 3.**
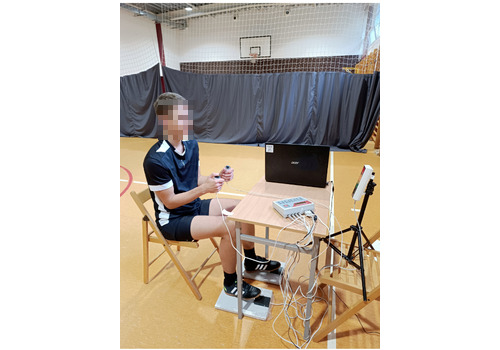
Measurement of cognitive efficiency: simple reaction time (SRT) testing using the Alfa-Electronics apparatus in adolescents with mild intellectual disabilities (n=8) at a special school in Poland (October 2023 to May 2024).

Cognitive efficiency was quantified using the Alfa-Electronics (Ustrzyki Dolne) standardized reaction time meter [43-46]. This hardware-based measurement provided an objective record of the latency (in ms) between a randomized stimulus and the participant’s motor response [43,44]. The SRT test serves as a fundamental metric for evaluating the efficiency of the central nervous system and sensorimotor coordination [17,47,48]. The device presented visual and auditory stimuli in a randomized, alternating order, and the participant was instructed to react as quickly as possible to either modality. The final SRT value represents the combined average latency across all randomized stimuli. The measurement procedure, featuring a participant performing the SRT test using the specialized apparatus, is illustrated in Figure 3. In alignment with previous research, this measurement protocol and the Alfa-Electronics apparatus have been validated, showing high internal consistency with a Cronbach α of 0.958 and a split-half reliability coefficient of 0.818 (*P*<.001) [49].

### Masking

Due to the nature of the immersive VR intervention, complete blinding of the participants and the PE instructor delivering the sessions was not feasible. However, to minimize assessment bias, a single-blind protocol was implemented for outcome evaluations. The pretest and posttest assessments (BOT-2 and SRT) were conducted by an independent examiner who was not involved in the execution of the 16-session VR intervention and remained unaware of the study’s specific intermediary training hypotheses. Furthermore, a single-blind procedure was maintained during data diagnostics, as all participant datasets were completely anonymized and coded prior to statistical analysis, ensuring the investigator remained masked to identity during processing.

### Psychometrics

The BOT-2 SF [37] is a standardized instrument, and also highly effective for populations with developmental challenges, demonstrating excellent internal consistency and superior test-retest reliability specifically in individuals with IDs [38]. The SF version was specifically selected for this study due to its high psychometric efficiency and its strong correlation with the complete form (*r*=0.80-0.87) [39,40].

Cognitive efficiency was quantified using the Alfa-Electronics (Ustrzyki Dolne, Poland) standardized reaction time meter [43-46]. This measurement protocol and the Alfa-Electronics apparatus have been validated, showing high internal consistency with a Cronbach α of 0.958 and a split-half reliability coefficient of 0.818 (*P*<.001) [49].

### Conditions and Design

The research followed a nonrandomized, single-group pretest and posttest pilot design involving a structured experimental manipulation. This specific design was selected to evaluate the initial feasibility and efficacy of the WISH and WON protocols within a naturalistic special education environment, where randomization into a control group was not practically feasible due to the limited total cohort of eligible students. To address concerns regarding the internal consistency of results in a small-scale study, a within-subject comparison was used. This approach effectively minimizes the confounding impact of high interindividual variability in motor and cognitive baselines, a common characteristic of the ID population, by using each participant as their own control. To ensure methodological rigor, all pretest and posttest assessments were strictly standardized, occurring under identical environmental conditions (same room, time of day, and examiners) using validated instruments. Furthermore, the 8-month duration of the 16-session intervention was strategically planned to facilitate the observation of stable neuro-motor adaptations, ensuring that the results reflect genuine developmental shifts toward normative thresholds rather than temporary performance fluctuations or novelty effects.

### Data Diagnostics

Planned data diagnostics included the exclusion of anticipatory errors in SRT measurements. Latencies shorter than 100 ms were defined as statistical outliers and excluded from the final analysis. Mean values were calculated from stable trials across the three measurement series to ensure data reliability. There were no missing data in this study (0%). All 8 participants completed all pretest and posttest assessments, which precluded the need for missing data imputation or the Missing Completely at Random test.

### Analytic Strategy

Statistical analysis was performed using Statistica 13.0 (TIBCO Software Inc.). Descriptive statistics, including the mean, median, and SD, were calculated for both motor proficiency and reaction time parameters. Due to the small sample size (n=8), nonparametric statistical methods were used to ensure the robustness of the findings. The Wilcoxon signed-rank test was used to compare pretest and posttest outcomes. The level of statistical significance was set at *P*<.05. To determine the magnitude of the intervention’s impact, effect sizes were calculated using the formula *r*=Z/**√** N. Effect sizes were interpreted according to Cohen criteria: 0.1 for a small effect, 0.3 for a medium effect, and 0.5 for a large effect.

## Results

### Participant Flow

**Figure 4.**
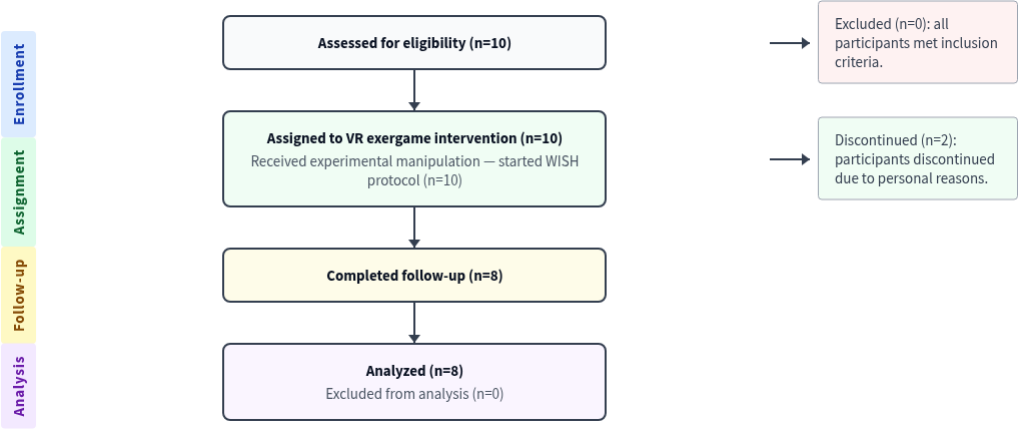
Flowchart of participants through the stages of the 16-session VR exergame intervention in adolescents with mild intellectual disabilities (n=8) at a special school in Poland (October 2023 to May 2024). VR: virtual reality; WISH: warm-up, imitation, settings, half-hour.

As illustrated in the participant flowchart (Figure 4), a total of 10 eligible students from the special school complex were initially assigned to the VR exergame intervention and commenced the training framework. Following the completion of the foundational WISH phase, 2 participants discontinued their participation due to personal reasons. The remaining 8 participants successfully proceeded to the WON phase, demonstrated 100% adherence, and completed all 16 scheduled training sessions. Consequently, the final analytical sample tracked from assignment through to the final posttest series consisted of 8 individuals.

### Sampling Procedures

Participants were recruited through a convenience sampling strategy at a single institutional setting (a special school complex in Poland). Out of the initial pool of eligible students approached within the facility, a 100% enrollment rate was achieved, resulting in 10 active participants entering the training framework. Data collection and empirical tracking were comprehensively conducted over an 8-month period from October 2023 to May 2024, ultimately yielding a final analytical sample of 8 adolescents after accounting for procedural attrition during the initial phase of the intervention.

### Statistic and Data Analysis

#### Missing Data

There were no missing data encountered at any assessment stage of this study (0% missing data frequency). All 8 participants included in the final cohort completed both the pretest and posttest measurement series in their entirety. Therefore, data imputation procedures were unnecessary, and missing completely at random data testing was precluded.

#### Statistical Assumptions and Software

Due to the exploratory nature and small size of the final analytical cohort (n=8), data distributions were assumed to be nonnormal, and nonparametric statistical procedures were used to maintain mathematical rigor. All inferential and descriptive computations were conducted using Statistica 13.0 software. The level of statistical significance for all corresponding hypothesis tests was set a priori at *P*<.05. Treatment fidelity was high, with 100% protocol adherence among the final sample under the uniform guidance of a single trained instructor.

#### Baseline Data

Baseline demographic and clinical characteristics for the final analytical sample (n=8) are summarized in Table 1. At the onset of the study (session 0), the participants exhibited significant motor proficiency deficits, with a group mean qualitative standing categorized as ’below average’ (mean 1.88, SD 0.64). All participants were efficiently handed and had no prior exposure to immersive VR environments.

**Table 1.** Baseline demographic and clinical characteristics of adolescents with mild intellectual disabilities (n=8) at a special school in Poland (October 2023).

Characteristic	Value (n=8)
Demographics	
Age (years), mean (SD; range)	17.63 (1.77; 15-20)
Sex, n (%)	
Male	3 (37.5)
Female	5 (62.5)
Clinical status, n (%)	
Diagnosis (mild intellectual disability)	8 (100)
Prior VR^a^ experience (none)	8 (100)
Baseline performance (pretest)	
BOT-2^b^ total point score, mean (SD; 95% CI)	69.25 (6.80; 63.56‐74.94)
BOT-2^b^ qualitative category, mean (SD)	1.88 (0.64)
SRT^c^ (ms), mean (SD; 95% CI)	426.50 (52.3; 382.78‐470.22)

a VR: virtual reality.

b BOT-2: Bruininks-Oseretsky Test of Motor Proficiency

c SRT: simple reaction time.

#### Primary Hypothesis: Motor Proficiency

The primary hypothesis assumed that the 16-session VR intervention would significantly improve global motor proficiency (BOT-2 total score). The Wilcoxon signed-rank test confirmed a statistically significant postintervention increase. The group’s mean total BOT-2 score rose from 69.25 (SD 6.80; 95% CI 63.56‐74.94; range 55‐74) to a posttest mean of 80.50 (SD 5.50; 95% CI 75.90‐85.10; range 72‐88). This change was statistically significant (*P*=.01; *r*=0.74) according to the Wilcoxon signed-rank test [Table 2].

**Table 2.** Pretest and posttest motor proficiency (BOT-2)^a^ outcomes in adolescents with mild intellectual disabilities (n=8) following a 16-session virtual reality (VR) intervention (October 2023–May 2024).

	Pretest (session 0), mean (SD; 95% CI)	Posttest (after session 16), mean (SD; 95% CI)	Difference, mean (*P* value)
BOT-2 total score (points)	69.25 (6.80; 63.56-74.94)	80.50 (5.50; 75.90-85.10)	+11.25 (.01)
BOT-2 qualitative category^b^	1.88 (0.64; 1.34-2.42)	2.88 (0.35; 2.59-3.17)	+1.00 (.008)
SRT^c^ (ms)	426.50 (52.3; 382.78-470.22)	392.10 (38.4; 359.98-424.22)	−34.40 (.02)

a BOT-2: Bruininks-Oseretsky Test of Motor Proficiency, Second Edition (Short Form). Total point score range: 0‐88.

b Descriptive categories mapped to a 5-point Likert scale: 1: well below average; 2: below average; 3: average (normative parity); 4: above average; 5: well above average. For individuals with intellectual disabilities, reaching Category 3 (average) represents the achievement of functional parity with typically developing peers.

c SRT:simple reaction time.

#### Secondary Hypothesis: Cognitive Efficiency (Reaction Time)

Objective measurements of neuro-cognitive efficiency (combined visual and auditory SRT) revealed a statistically significant improvement in response speed. The mean SRT shortened by 34.40 ms, decreasing from a baseline of 426.50 (SD 52.3; 95% CI 382.78‐470.22) ms to 392.10 (SD 38.4; 95% CI 359.98‐424.22) ms at the final assessment (*P*=.02) (Table 2). Beyond the improvement in average response speed across both visual and auditory modalities, participants demonstrated enhanced neuro-cognitive stability. This was evidenced by a reduction in response variability and a narrowed range between minimum and maximum reaction times compared to the initial measurements.

### Exploratory Hypotheses and Estimates

The first exploratory analysis investigated whether the numerical increase in BOT-2 point scores translated into a meaningful clinical shift in the participants’ descriptive motor categories (eg, moving from a “below average” classification to an “average” classification). Analysis of the 5-point standardized qualitative proficiency scale revealed a significant upward shift. The group mean increased by exactly one full descriptive category, rising from 1.88 (SD 0.64; 95% CI 1.34‐2.42) at baseline to 2.88 (SD 0.35; 95% CI 2.59‐3.17) postintervention (*P*=.008, *r*=0.89). Practically, this indicates that 75% of the participants (6 out of 8 individuals) successfully transitioned from a “below average” motor standing to “average” functional parity, reaching the normative motor levels expected for their typically developing peers.

The second exploratory analysis examined whether the VR training framework improved the consistency of the participants’ focus and reaction times, rather than just their raw speed. In individuals with IDs, high variability between the fastest and slowest reactions often indicates attention deficits or executive dysfunction. Posttest evaluations of the SRT data revealed a marked reduction in this variability. Specifically, the statistical range, the gap between the individual’s minimum (fastest) and maximum (slowest) response latencies across the measurement trials, was substantially constricted compared to the initial baseline metrics. This narrowing of the response range indicates that following the intervention, the participants not only reacted faster but also executed the cognitive tasks with greater neurological stability, consistency, and sustained attention.

## Discussion

### Principal Findings

The primary objective of this pilot study was to evaluate the feasibility and preliminary effectiveness of a structured VR exergame intervention, governed by the WISH and WON protocols, in enhancing motor proficiency and cognitive processing speed in adolescents with mild ID. The findings provide encouraging support for both the primary and secondary hypotheses, indicating that a systematically guided digital intervention can elicit positive shifts in global motor coordination and balance while accelerating simple reaction times to sensory stimuli [50]. Furthermore, exploratory analyses revealed an enhanced stability in neuro-cognitive responses and a qualitative migration of participants toward normative parity, suggesting that technology-driven frameworks can successfully support stable sensorimotor adaptations and reduce intra-individual variability in youth with neurodevelopmental conditions [50].

### Comparison With Prior Work

These outcomes strongly contribute to the growing consensus regarding the clinical utility of immersive environments in special education and neurorehabilitation [33]. When compared to traditional, long-term sports programs, such as hockey, basketball, or swimming, which often require extensive multimonth timelines or trigger high attrition rates due to social isolation, the intensive digital framework evaluated here offers a highly motivating and time-efficient alternative [9,51,52]. The observed trends in postintervention coordination approached established western developmental benchmarks [53] and compared favorably against regional normative data reported for youth with ID [6].

However, this study observed broader global motor improvements than those reported in nonimmersive digital interventions [13], potentially due to the highly rhythmic, full-body immersive nature of the chosen exergame [32]. While some earlier studies suggested that 12-month programs might be necessary for individuals with severe ID [51], our preliminary data corroborate emerging evidence that shorter, intensive game-based interventions can effectively elicit measurable neurocognitive and proprioceptive changes in mild ID cohorts [17,54]. Additionally, the structured protocol used here offers a promising, time-efficient alternative to longer traditional sports interventions [52]. Such results highlight VR active games and digital exergames as noninvasive, accessible tools that can enhance physical literacy domains and movement skills while encouraging broader participation in physical activities [55,56].

A critical insight from this study lies in the interaction between immersive interfaces and motor skill acquisition. Prior work demonstrated that while stationary bike-based VR exergames improve locomotor skills, they fail to enhance object control or ball skills because basic body redirection or hand controllers do not provide sufficient sensorimotor feedback for complex manipulation [23]. Similarly, recent longitudinal evidence indicates that unguided fully immersive VR training yields limited skill transfer to real-world tasks or generalized reaction times [24]. This study directly addresses these challenges by incorporating the WISH and WON protocols to provide structured instructional guidance [29]. By scaffolding the user’s experience, these protocols appeared to facilitate superior motor control and response quickness, aligning with recent clinical trials which show that task-oriented VR active games systematically enhance predictive internal modeling and visuomotor adaptation in children with developmental coordination challenges [55,57]. Although updating internal models often involves persistent compensatory errors and motor variability [57], the combination of immersive stereoscopic visualization and structured pedagogical scaffolding appears to minimize cognitive load, helping users form and generalize more accurate predictive motor plans [4,5,57].

Mechanistically, combining computerized cognitive training with motion-based digital interactions provides a highly effective multisensory environment that substantially promotes visual-motor integration and global motor functions in individuals with intellectual challenges [58]. By leveraging adaptive technology, such integrated cognitive-motor interventions expand the complexity of the perceptually enriched world users interact with, actively accelerating general visual perception, visual-motor speed, and spatial relations [58].

Furthermore, immersive therapeutic role-playing setups have been verified to yield statistically significant improvements in concentration scores and active engagement, highlighting the profound clinical value of tailored hand-eye coordination training in game-based environments [59].

The positive changes in motor independence and cognitive engagement observed in this study reinforce the theoretical foundations of Self-Determination Theory within digital learning [36]. Recent qualitative evidence demonstrates that when immersive VR programs are paired with facilitative, nondirective professional support, they function as psychologically safe environments that significantly bolster self-determination, personal agency, and action-control beliefs [26]. While such agency-building effects have primarily been documented in aging adults with ID [26], this study extends this novelty to an adolescent cohort, confirming that providing autonomy support and structured containment can successfully foster autonomous gameplay and perceived competence in younger populations [26,29,36]. This is highly consistent with contemporary findings from international special education initiatives, which emphasize that structured VR-based motor skill programming yields high usability, robust pedagogical effectiveness, and an emotionally secure student experience [60]. Similarly, the incorporation of interactive mobile architectures and augmented reality in physical education has been shown to alleviate cognitive friction, thereby accelerating gross motor skill mastery in elementary and secondary school environments [51,61,62].

To address the crucial “so what?” question regarding the real-world value of this project, the simultaneous improvements documented in motor proficiency and visual-motor speed carry profound practical benefits for the daily independent living and personal safety of adolescents with mild ID. Translating laboratory-derived metrics into concrete functional autonomy means that these adolescents can navigate their everyday physical environments with greater security. Specifically, the significant acceleration in SRTs directly translates into enhanced pedestrian safety, allowing individuals to react faster to sudden real-world hazards, such as an oncoming vehicle when crossing a street. At the level of daily routines, the refinement of fine and gross motor mastery directly supports the performance of essential activities of daily living, such as handwriting, object manipulation, or tying shoes [58]. Crucially, contemporary locomotor evidence underscores that VR protocols incorporating enriched sensory feedback and progressive task difficulty possess high ecological validity, proving that behavioral modifications learned while stepping in place in a virtual setting successfully transfer to actual walking behaviors in a real environment [63]. By fine-tuning movement patterns and reducing spatial margins without increasing the danger of physical contact, this structured training diminishes the real-world risk of accidental tripping and catastrophic falls [63,64]. This protective mechanism is further reinforced by target-oriented exergaming frameworks which demonstrate that digital sensorimotor tracking significantly enhances shoulder joint position sense and lower-extremity neuromuscular functionality during functional sit-to-stand activities [54]. By stabilizing postural control and peripheral proprioceptive awareness, such interventions directly address the core integration deficits typical of neurodevelopmental conditions [54].

However, long-term follow-up models in adapted physical activity warn that while structured interventions markedly accelerate motor milestones, these acquired motor adaptations significantly regress if continuous participation is discontinued, yielding a substantial loss of the acquired gains specifically in vital domains like bilateral coordination and balance [65]. This clinical reality underscores the absolute necessity of transitioning this VR protocol into a continuous, long-term institutional routine rather than a transient laboratory experiment [65].

From an institutional implementation standpoint, this structured exergaming intervention provides a highly practical, standardized, and scalable solution for special education frameworks and therapeutic workflows. Special schools frequently struggle with compromised student motivation and high attrition rates in traditional PE sessions, where conventional exercises are often perceived as monotonous, overly rigorous, or exclusionary. This intervention directly overcomes this motivational barrier by offering an attractive alternative to boring PE, using the inherent appeal of gamification, child-friendly characters, and multimodal reward structures to secure a safe and emotionally supportive environment [58]. Indeed, recent pediatric exercise data confirm that integrating novel exergaming guided by objective, real-time physiological metrics, such as visually targeted heart rate zones, yields flawless compliance, keeping children deeply physically challenged and intrinsically motivated without experiencing technological boredom [66].

For educational specialists and oligophrenopedagogues, this structured digital design represents a ready-to-use asset. The system offers ease of replication, high safety standards, and robust engagement metrics [66]. The specific deployment of the WISH and WON instructional scaffolds directly mitigates operational risks; spatial safety mapping indicates that under unguided conditions, virtual collisions occur predominantly on the “trailing” or backward-facing side of the user’s body due to localized visual-spatial gaps [63]. By providing structured containment, our protocols ensure whole-body sensory recalibration and comprehensive motor planning.

Nevertheless, school-based deployment strategies must remain flexible; multitask sensorimotor evidence reveals that while active digital systems trigger substantial functional gains, neurodivergent cohorts frequently display a “high-gain yet unstable” learning curve, characterized by significant intragroup and trial-by-trial performance volatility [67]. By leveraging these structured pedagogical scaffolds, special education professionals can successfully compensate for these psychomotor fluctuations, stabilizing long-term motor skill acquisition over time [67].

### Limitations

Several limitations must be acknowledged to contextualize the generalizability and external validity of these exploratory findings.

First, as a pilot investigation, the small sample size restricts statistical power and limits immediate generalizability to the broader population of adolescents with ID.

Second, the single-group pretest and posttest architecture lacks a parallel control group, which inherently limits the ability to draw definitive causal claims regarding the standalone efficacy of the VR exergame independent of natural maturation or external factors.

Third, the wide chronological age bracket encompassing late adolescence involves varying degrees of biological and neuromuscular maturation, which may have acted as a confounding variable influencing individual motor trainability [4,53].

Fourth, while the BOT-2 SF is highly sensitive for identifying motor delays, its lower specificity for mapping advanced athletic developments implies that the observed changes primarily reflect the closing of developmental gaps rather than high-tier athletic skill acquisition [37-40]. In consideration of reporting guidelines regarding the precision of measurement protocols, it should be noted that the streamlined design of this abbreviated tool focuses primarily on broad functional changes rather than highly granular, microlevel adjustments. While this SF protocol is exceptionally efficient and well-validated for capturing macrolevel developmental shifts in youth with ID [37-40], its structural brevity means it might not register minor, step-by-step psychomotor fluctuations, thus presenting a highly stable and conservative estimate of the participants’ actual motor progress.

Fifth, although the intervention demonstrated robust psychometric gains, this study did not directly evaluate the long-term ecological transfer of these motor skills to unstructured, real-world daily living activities, a limitation that mirrors ongoing transfer challenges highlighted in recent neurodevelopmental literature [24,26].

Finally, because immersive technology was a novelty in the local educational setting, the long-term retention of these sensorimotor improvements remains uncertain once the initial novelty effect subsides, a phenomenon frequently cited as a barrier in digital health feasibility models, crossover setups, and scoping reviews of exergaming compliance [56,68,69].

### Conclusions

In conclusion, this pilot study provides valuable preliminary evidence that structured pedagogical frameworks, such as the WISH and WON protocols, can successfully transform commercial immersive exergames into effective clinical and educational assets for addressing motor and cognitive delays in adolescents with mild ID. These findings indicate that integrating autonomy-supportive digital environments into physical education curricula offers a motivating, stigma-free avenue to improve physical literacy and cognitive processing stability [36,56]. While these early-stage results are highly encouraging, further research using larger, randomized controlled trials with multicenter recruitment and long-term longitudinal follow-ups is strictly necessary to validate these trends and establish standardized guidelines for digital health interventions in special education [68,69].

## Abbreviations

BOT-2: Bruininks-Oseretsky Test of Motor Proficiency
ID: intellectual disability
PE: physical education
SF: short form
SRT: simple reaction time
VR: virtual reality
WISH: warm-up, imitation, settings, half-hour
WON: warm-up, objective evaluation, no problem!
